# Predicting the immune microenvironment and prognosis with a anoikis - related signature in breast cancer

**DOI:** 10.3389/fonc.2023.1149193

**Published:** 2023-07-04

**Authors:** Xiuqing Lu, Qi Yuan, Chao Zhang, Sifen Wang, Weidong Wei

**Affiliations:** ^1^ Department of Breast Surgery, Sun Yat-Sen University Cancer Center, State Key Laboratory of Oncology in South China, Collaborative Innovation Center for Cancer Medicine, Guangzhou, China; ^2^ State Key Laboratory of Oncology in South China, Collaborative Innovation Center for Cancer Medicine, Sun Yat-Sen University Cancer Center, Guangzhou, China; ^3^ Department of Radiation Oncology, Sun Yat-Sen University Cancer Center, State Key Laboratory of Oncology in South China, Collaborative Innovation Center for Cancer Medicine, Guangzhou, China

**Keywords:** anoikis, breast cancer, prognosis, immune infiltrates, immunotherapy

## Abstract

**Background:**

Tumor heterogeneity is widely recognized as a crucial factor impacting the prognosis of breast cancer (BC) patients. However, there remains an insufficient understanding of the underlying impact of anoikis on the prognosis of BC patients.

**Methods:**

The researchers utilized the TCGA-BRCA dataset to screen and analyze the differentially expressed genes of anoikis-related genes (ARGs) in BC and normal breast tissue. Prognostic gene signatures were established through univariate, LASSO, and multivariate Cox regression analyses. These signatures were evaluated using Kaplan-Meier curve and receiver operating characteristic (ROC) analyses, resulting in the development of an anoikis-related index (ACI). The training dataset was TCGA-BRCA, while METABRIC and GSE96058 were used for external validation. Additionally, nomograms were developed by combining risk scores and clinical parameters, enabling gene set enrichment analysis (GSEA) and tumor immunoassay. Furthermore, an exploration of small molecule compounds was conducted to identify potential therapeutic benefits.

**Results:**

A six-gene anoikis-related signature was constructed, which divided BC patients into high- and low-ACI groups based on median ACI scores. The ACI accurately predicted prognosis and acted as an independent prognostic factor for BC patients. Patients in the high-ACI group exhibited poorer overall survival (OS) across all cohorts and showed more severe clinical manifestations compared to the low-ACI group. The study also explored the potential impacts of anoikis on immune cells infiltrating tumors, immune checkpoints, growth factors, and cytokine levels. Additionally, the potential implications of anoikis in BC immunotherapy were discussed, along with highlighting small molecule compounds that could offer therapeutic benefits.

**Conclusions:**

Anoikis was found to hold significant prognostic value in breast cancer, providing a novel approach for managing patients with different prognoses and implementing more precise immunotherapy strategies.

## 1 Introduction

Breast cancer is a prevalent form of cancer globally and ranks as the second most common cause of cancer-related deaths in women (1). Despite progress in early detection and treatment, evidenced by a 38% reduction in breast cancer mortality, the overall prognosis for breast cancer patients remains unfavorable (2). The heterogeneity of breast cancer greatly increases the difficulty of treatment. Therefore, identifying novel prognostic biomarkers for breast cancer holds immense significance in improving treatment outcomes and predicting patient prognosis. The extracellular matrix (ECM) serves as a three-dimensional scaffold that supplies essential biochemical cues for tissue formation and regulates cell proliferation, migration, differentiation and survival (3). Anoikis is a form of programmed cell death characterized by the detachment of cells from their natural extracellular matrix and subsequent apoptosis upon losing contact with adjacent cells or ECM (4). Consistent with other apoptotic modes, anoikis also occurs by interfering with mitochondria or activating cell death receptors (5). The exfoliated cells can be efficaciously removed by anoikis, preventing them from reattaching to a newly created substrate and proliferating (6). Therefore, anoikis is considered physiological processes related to development and tissue steady-state (7). In theory, anoikis can inhibit the metastasis of cancer cells. However, cancer cells are of insensitivity to anoikis and do not need to adhere to the ECM to subsist and reproduce (8). Increasing studies have confirmed that anoikis-related genes (ARGs) play a crucial role in the occurrence and development of tumours over the years. At present, anoikis resistance has become a marker of cancer cell invasion, metastasis, drug resistance and recurrence.

It is well known that the tumour microenvironment (TME) can identify and eliminate cancer cells, however, cancer cells can recruit immunosuppressive cell populations and downregulate tumour immunogenicity to regulate the host immune system and escape immune surveillance (9, 10). The secretion of growth factors in the tumour microenvironment, such as vascular endothelial growth factor (VEGF) and transforming growth factor-β (TGF-β), can impede the immune response, thereby inhibiting the effect of immunotherapy (11, 12). Cytokines can limit the growth of tumour cells either by immediate antiproliferative or proapoptotic activity or mediately by provoking the cytotoxic activity of immune cells against tumour cells (13). Previous studies have shown that ARGs are more likely to promote cancer immunosuppression (14–16). However, the role of anoikis in tumour immunity is less studied, and whether it can regulate cytokines and growth factors remains to be further elucidated.

Although there are now many independent studies linking ARGs to BC, prognostic targets based on ARG are rarely analyzed (16–18). In this work, we explored the prognostic implications of ARGs and established a signature based on ARG signaling, and discussed its clinical significance in BC patients. Furthermore, we explored potential correlations between the signature and TME landforms. Our analysis offers novel insights that may inform future research into anoikis and immunotherapy approaches for BC treatment.

## 2 Materials and methods

### 2.1 Data acquisition

We acquired RNA expression data, CNV files, somatic mutation data and corresponding clinical pathological information for breast cancer from The Cancer Genome Atlas (TCGA, https://portal.gdc.cancer.gov/), the Molecular Taxonomy of Breast Cancer International Consortium (METABRIC, https://www.bccrc.ca/dept/mo/), and GSE96058 in the GEO repository. Obtaining clinical parameters and normalized gene expression data through the utilization of these data sources.

### 2.2 Obtention of anoikis-related genes

We obtained 434 anoikis-related genes (ARGs) from the GeneCard database (https://www.genecards.org/) (15). The 434 ARGs mentioned above were hybridized with the entire set of genes in TCGA-BRCA, METABRIC, and GSE96058 datasets, from which 338 overlapping ARGs were identified and selected for subsequent analysis.

### 2.3 Identification of DEGs and functional enrichment analysis

Differentially expressed genes (DEGs) between tumor tissues and contiguous normal areas in TCGA-BRCA cohort were analysed by using the “limma” package with criteria of |log2-fold change (FC)| ≥ 1 and p-value< 0.05. Subsequently, we employed the “clusterProfiler” package to perform a GO analysis based on these identified DEGs (19).

### 2.4 Construction ARGs-based prognostic signature in breast cancer

Univariate Cox risk regression analysis in TCGA-BRCA and METABRIC databases was used to generate ARG related to OS (p<0.05). To establish the statistical prognostic characteristics, we conducted a Lasso Cox regression analysis in the TCGA-BRCA set for the overlapping ARGs that were associated with OS. According to the predictive model, the Anoikis-correlation index (ACI) could be exported using the following formula:


ACI=∑Expression of Each ARG∗Corresponding Regression Coefficient

We categorized all breast cancer patients into two groups - high ACI and low ACI, based on the median value of the Anoikis-correlation index (ACI). Furthermore, we compared the clinicopathological features of patients belonging to different ACI groups in the three datasets.

### 2.5 Creation and assessment of anoikis-correlated clinical nomogram

The “survival” package was used to combine clinical data of TCGA-BRCA patients and ACI for independent prognostic analysis. Moreover, according to the results above, an anoikis- related clinical nomogram that incorporated clinicopathological characteristics to predict the clinical outcome of cases was constructed through the “rms” and “regplot” R packages (20). To estimate the acceptable prognosticative discrimination of the nomogram, the calibration curve (21), time-dependent ROC curves and the decision curve analysis (DCA) plot were depicted for breast cancer patients.

### 2.6 Gene set enrichment analysis within two ACI groups

GSEA was used to detect the biological pathways and immunological activity related to ARGs based on the Hallmark and C7gene set v7.4 (22). Enriched genesets were chosen as the reference molecular signature databases, and |NES| > 1.5 and FDR q-value< 0.05 were considered as statistical significance.

### 2.7 Correlation of tumor-immune microenvironment and ACI

We used the “estimate” Rpackage to numerate the immune score and matrix score for each specimen in the TCGA-BRCA cohort (23). Assess the proportion of 22 kinds of immune cells in TME of each sample through CIBERSORT algorithm in R software (24). The Wilcoxon test was applied to compare the expression difference of several immune checkpoints, cytokines, and growth factors between high- and low-ACI subgroups to predict the potential impact of anoikis on immunotherapy (25, 26).

### 2.8 Cell lines

MCF-10A, MCF-7, HCC1599, MDA-MB-231 and SK-BR-3 were obtained from ATCC (American Type Culture Collection) and SUM159PT was supplied by Aster-and Bioscience. The cells were cultured in Dulbecco’s Modified Eagle Medium (DMEM) supplemented with 10% fetal bovine serum (FBS) and maintained in a CO2 incubator at 37°C. The MycoSEQ™ Mycoplasma Detection Kit (#4460623, Thermo Fisher Scientific) was utilized to detect mycoplasma contamination in the cell culture. Before we began our experiments, a short tandem repeat (STR) profile test was used to formally qualify the cell lines.

### 2.9 RNA isolation and quantitative real-time PCR analysis

Thermo Fisher Scientific’s TRIzol kit was used to extract the RNA. Two microgram RNA was mixed with RNase-free DNase, and then reversely transcribed into cDNA. After combining two micrograms of RNA with RNase-free DNase, cDNA was produced using reverse transcription. On the CFX96 Real-Time PCR Detection System (Bio-Rad), qPCR using SYBR premix Ex Taq (Takara) and one microliter of cDNA was performed. To determine the relative expression level, the Ct value of target genes was contrasted with that of glyceraldehyde-3-phosphate dehydrogenase (GAPDH).

### 2.10 Cancer cell line encyclopedia analysis

In order to further verify the ARGs mRNA expression, we passed the CCLE database (https://portals.broadinstitute.org/ccle) to further explore their expression levels in breast cancer cell lines. CCLE is a project aimed at characterizing the gene expression and genomic alterations in a large panel of human cancer cell lines (27). An RNA sequencing technique was chosen to search for expressions of ACI members in different breast cancer cell lines (28, 29).

### 2.11 DNA methylation of the anoikis-related genes

MethSurv (https://biit.cs.ut.ee/methsurv/) is a web-based tool for multivariate survival analysis using DNA methylation data, includes 7358 methylomes from 25 different human cancers (30). We employed MethSurv to investigate the expression and prognostic implications of individual CpG methylation in signature-contained ACIs in BC. In this analysis, we utilized MethSurv to conduct survival analysis with the “best” cut-off point option and obtained the cut-off point with the highest HR as output (31, 32).

### 2.12 Identification of potential compounds targeting the high-ACI group BC

The Connectivity Map (CMap) database is a publicly available database that enables the identification of relationships between gene profiles, drug sensitivity, and disease states (33, 34). The CMap data can be accessed and downloaded for free at https://clue.io. We utilized the R package “limma” to discover the DEGs between high- and low-ACI group breast cancer. Upon eliminating the DEG repetitions, we obtained a unique up- and down-regulated gene profile, which was subjected to further analysis by inputting the top 200 genes (100 upregulated and 100 downregulated) into the CMap database. Following this, we selected compounds in BC cell lines as potential drugs targeting BC in the high-ACI group (35).

### 2.13 Statistical analysis

In our research, all statistical analyses were performed with R4.2.0. For nonparametric data, the Wilcoxon test was applied to comparisons between two independent samples, and the Kruskal-Wallis test was applied to comparisons among multiple samples. The Kaplan-Meier survival curve and log-rank test were applied to analyze overall survival (OS) among different groups of BC patients. P-value< 0.05 was regard as statistical significance (*p-value< 0.05; **p-value< 0.01; ***p-value< 0.001).

## 3 Results

### 3.1 Identification of ARGs associated with prognosis in BC patients

**Figure 1:**
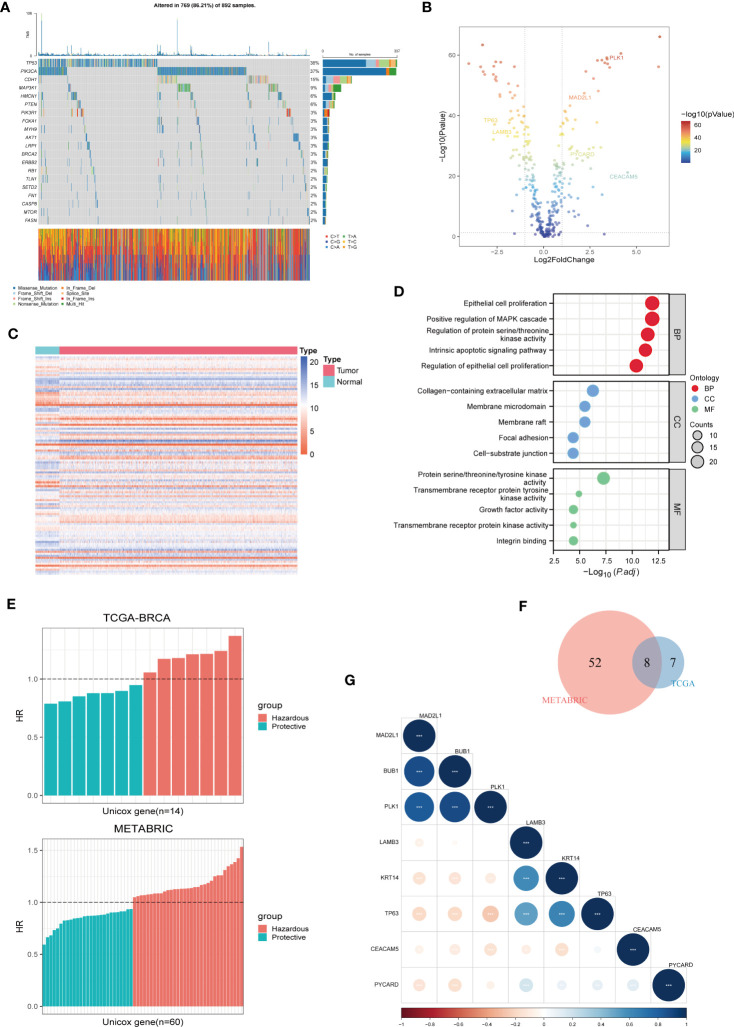
Identification of Prognostic ARGs in BC Patients. **(A)** A somatic mutation waterfall plot demonstrating the prevalence of driver mutations in ARGs across TCGA-BRCA cohort. **(B, C)** A volcano and a heatmap plot were used sequentially to display the differentially expressed ARGs between normal and tumor tissues in TCGA-BRCA. **(D)** GO enrichment analyses of differentially expressed ARGs. **(E)** Univariate Cox analysis was used to screen prognostic ARGs separately in TCGA-BRCA and METABRIC. **(F)** 8 prognostic ARGs were investigated using the Veen diagram. **(G)** A correlation matrix plot was used to reveal the correlation characteristics among the 8 prognostic ARGs in TCGA-BRCA.

First, we evaluated the somatic mutation incidence in 338 ARGs in the TCGA-BRCA cohort, and the waterfall plot shows the top 20 genes with the highest somatic mutation rates. As shown in **Figure 1A**, the highest mutation frequency distributed in TP53 (38%) and PIK3CA (37%). In addition, we compared the ARGs’ mRNA expression levels of breast cancer samples and adjacent normal tissues in TCGA-BRCA cohort with |log2FC| > 1 and FDR< 0.05 as thresholds. A total of 110 DEGs were found, of which 56 were upregulated and 54 were downregulated in tumours, and the results were shown by a volcano plot (**Figure 1B**) and a heatmap (**Figure 1C**). Furthermore, the biological functions of these DEGs were revealed by GO enrichment analysis (**Figure 1D**). As expected, these DEGs are clustered in the signal pathway related to epithelial cell proliferation and regulation protein serine/threonine/tyrosine kinase activity, indicating that ARGs were closely connected with carcinogenesis and development of BC. In addition, to determine the prognostic ARGs of breast cancer, we conducted univariate Cox regression analysis of DEGs in the TCGA-BRCA and METRIC cohorts (**Figure 1E**). After analysis, 14 and 60 available OS-related genes were obtained respectively, and the above results were crossed to include 8 overlapping genes (MAD2L1, BUB1, PLK1, LAMB3, KRT14, TP63, CEACAM5, and PYCARD) (**Figure 1F**). Subsequently, we revealed the correlated features among 8 eligible ARGs with correlation network graphs (**Figure 1G**).

### 3.2 Development and construction of anoikis-related prognostic signature

**Figure 2:**
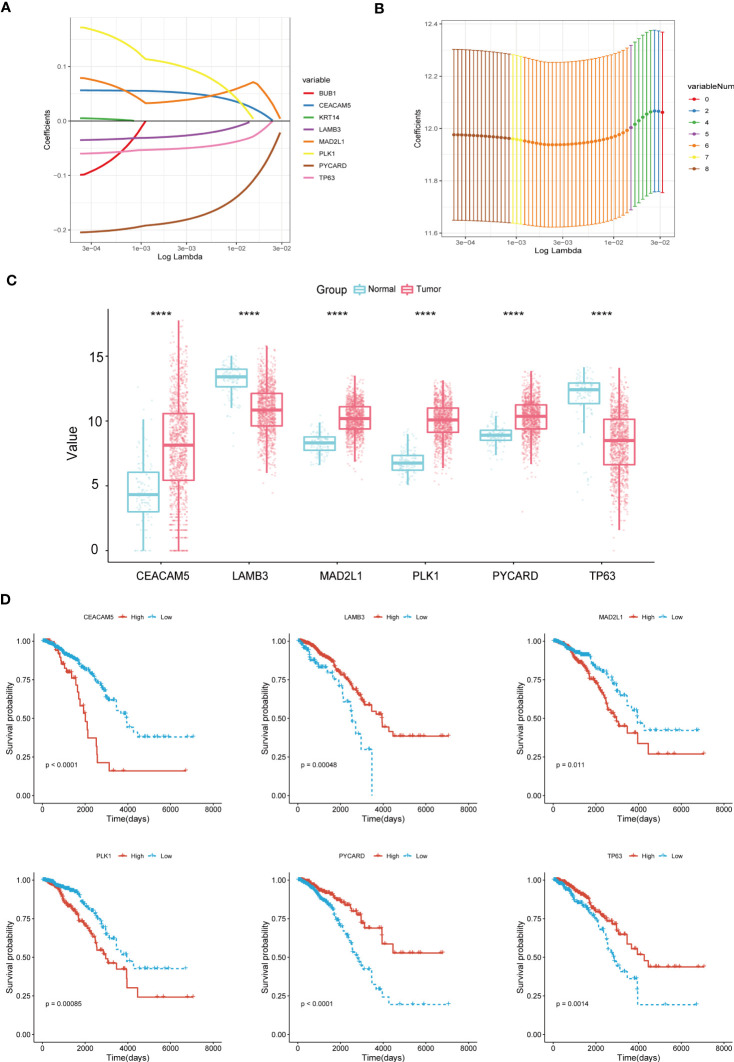
Construction of Anoikis-Related Prognostic Signature. **(A, B)** LASSO Cox regression analysis to filtrate 6 optimal prognostic ARGs for the signature. **(C)** Boxplots illustrating the mRNA expression levels of 6 signature-contained ARGs in the training cohort. **(D)** K-M survival curves of OS according to expression levels of 6 ARGs in the training cohort. ****p< 0.0001.

**Figure 3:**
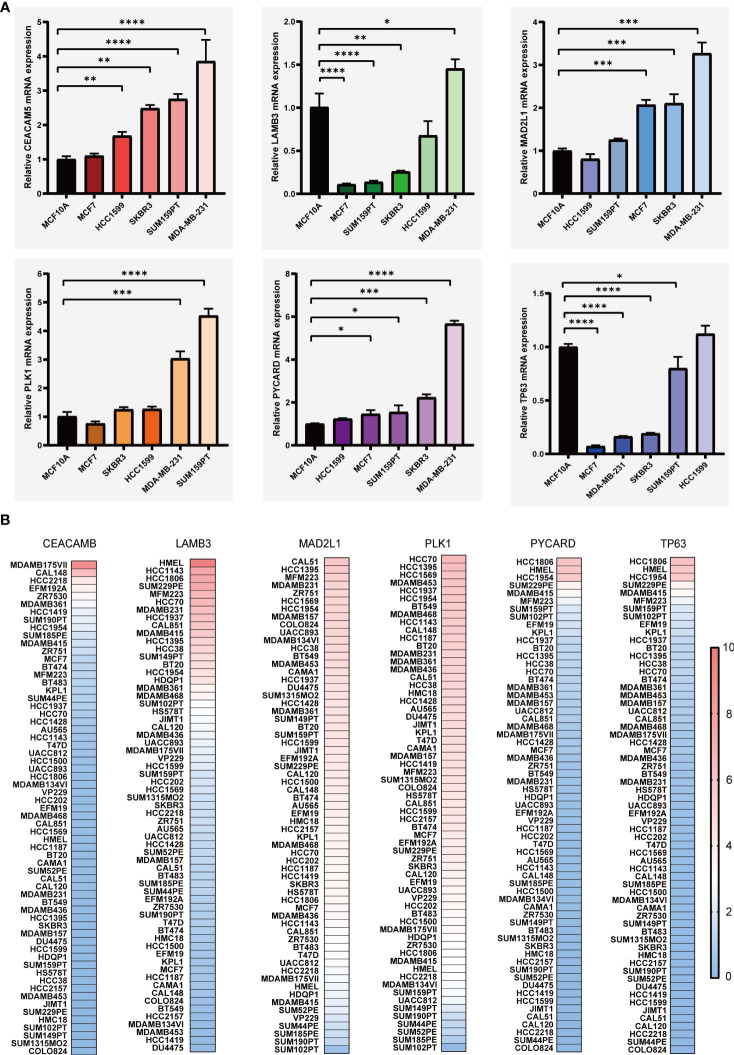
The levels of mRNA expression for ARGs. **(A)** qRT-PCR analyses of ARGs expression in human breast cancer cell lines and a normal mammary epithelial cell line. **(B)** Expressions of ARGs in different breast cancer cell lines from the CCLE database. *p< 0.05**; p< 0.01; ***p< 0.001; ****p< 0.0001.

We carried out the LASSO cox regression analysis of 8 eligible ARGs of BC patients in the TCGA-BRCA dataset, and tapped 6 central genes for establishing prognostic signature, namely Anoikis-Correlation Index (ACI) (**Figures 2A, B**), including MAD2L1, PLK1, LAMB3, TP63, CEACAM5 and PYCARD. In order to study the expression level and independent predictive ability of each feature gene, the mRNA expression level in tumour tissues and adjacent tissues was shown by boxplots (**Figure 2C**) and the correlation between ARGs expression and OS was shown by K-M survival curves (**Figure 2D**). Our results indicated that the mRNA expressions of CEACAM5, MAD2L1, PLK1, and PYCARD are significantly elevated in BC tissues, whereas LAMB3 and TP63 expressions are noticeably reduced. In terms of OS analysis, high expression levels of CEACAM5, MAD2L1, and PLK1 and downregulated expression of LAMB3, PYCARD, and TP63 were strongly linked to poor prognosis in BC patients, further supporting the selection of ARGs. Additionally, we assessed the mRNA expression levels of ACI members in prevalent human BC cell lines, such as MDA-MB-231, MCF-7, SK-BR-3, SUM159PT, and HCC1599 (**Figure 3A**). Our findings demonstrated that the expression levels of CEACAM5, LAMB3, MAD2L1, PLK1, PYCARD, and TP63 in most human BC cell lines matched tissue expression levels in the database, as compared to the mammary epithelial cell line MCF-10A. Moreover, the analysis of the CCLE dataset indicated varying mRNA expressions of ACIs members containing signatures in breast cell lines (**Figure 3B**). DNA methylation, as a form of epigenetic modification, plays a significant role in the pathogenesis of various cancers (36). Therefore, in our study, we utilized MethSurv to investigate the clustering heatmap and prognostic value of the DNA methylation expression levels of ACI members in BC (**Supplement Figure 1**). DNA methylation expression levels concluded that cg07168232 from LAMB3 and cg01965475 from PLK1 had the significant prognostic value (p-value< 0.05) in BC. Ultimately, the anoikis-correlation index (ACI) was established: ACI = Expression of CEACAM5*0.05275451 +Expression of LAMB3*-0.02805108 + Expression of MAD2L1*0.03686359 + Expression of PLK1*0.10316860 + Expression of PYCARD*-0.18480265+ Expression of TP63*-0.05094233

### 3.3 Validation of ARGs signature

**Figure 4:**
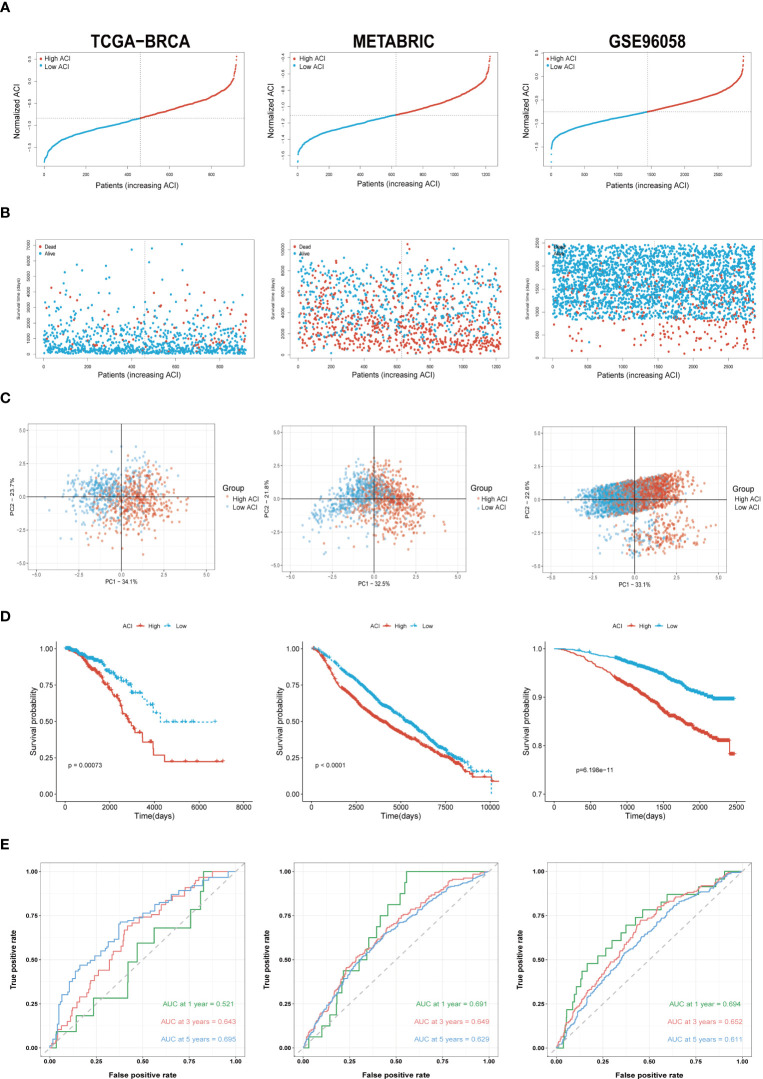
Validation of ARGs Signature. **(A)** The layout of ACI scores in the TCGA-BRCA, METABRIC and GSE96058 datasets. **(B)** Variations in BC patient mortality are being paralleled by an increase in ACI. **(C)** Analysis of PCA between high- and low-ACI groups. **(D)** K-M curves for the OS of BC patients in high- as well as low-ACI groups. **(E)** The ACI-only model’s ROC curves for estimating BC patients’ 1-, 3-, and 5-year OS.

Based on the median ACI in each dataset, BC patients were independently categorized into high-ACI and low-ACI subgroups, and the ACI was standardized to ensure the data and plots intuitionistic (**Figure 4A**). As predicted, in all datasets, as ACI values increased, more BC patients died (**Figure 4B**). Simultaneously, we show the distribution law of the high- and low-ACI group in two-dimensional graphs by principal component analysis (PCA) (**Figure 4C**). Just as the K-M curve shows, the ACI-high BC patients have a poor prognosis among all queues (**Figure 4D**). Further, the AUC values of ACI in predicting the breast cancer patients’ OS were 0.521 at 1 year, 0.643 at 3 years and 0.695 at 5 years (**Figure 4E**). We performed the same analysis on the two validation sets to further validate the prognostic value of ACI.

### 3.4 Correlation between ACI and clinicopathological features

**Figure 5:**
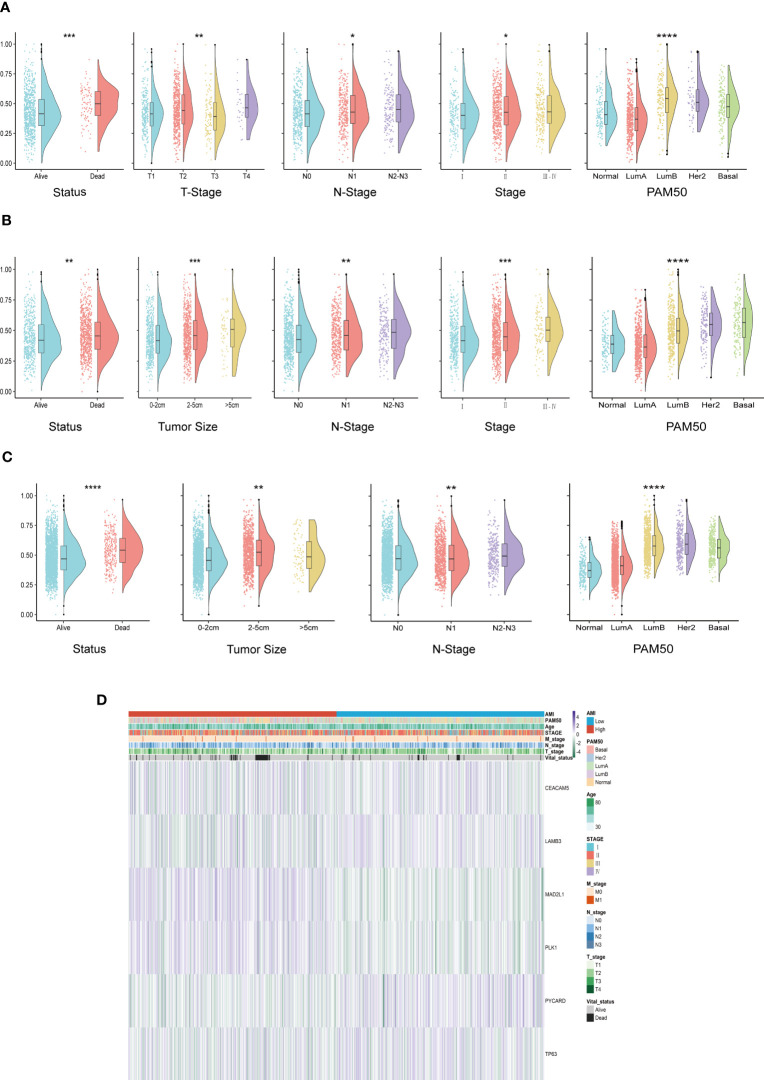
Systematic dissection of ACI and clinical parameters in BC patients. **(A-C)** The violin plots to demonstrate the relevance between ACI and diverse clinical-pathological traits of BC patients in TCGA-BRCA **(A)**, METABRIC **(B)** and GSE96058 **(C)**, respectively. **(D)** Relevance heatmaps of signature-included ARGs and clinical-pathological traits in datasets of TCGA-BRCA. *p< 0.05; **p< 0.01; ***p< 0.001; ****p< 0.0001.

In order to explore whether ACI can predict common clinical indicators, the correlation between ACI and clinical characteristics has been further studied. In the TCGA-BRCA queue, there are significant differences in T stages, N stages, survival status, PAM50 subtypes and AJCC stages between two groups (p value<0.05) (**Figure 5A**). Likewise, in the METABRIC cohort, significant variations were confirmed between patients with high ACI levels and various clinical parameters, such as poorer survival status, larger tumor size, and increased metastatic lymph nodes (**Figure 5B**) and in the GSE96058 cohort (**Figure 5C**). We used heatmaps to show the correlation analysis between genes contained in ACI and clinical characteristics in the TCGA-BRCA dataset (**Figure 5D**), METABRIC dataset (**Supplement Figure 2A**) and GSE96058 dataset (**Supplement Figure 2B**).

### 3.5 Nomogram formulation based on ACI signature

**Figure 6:**
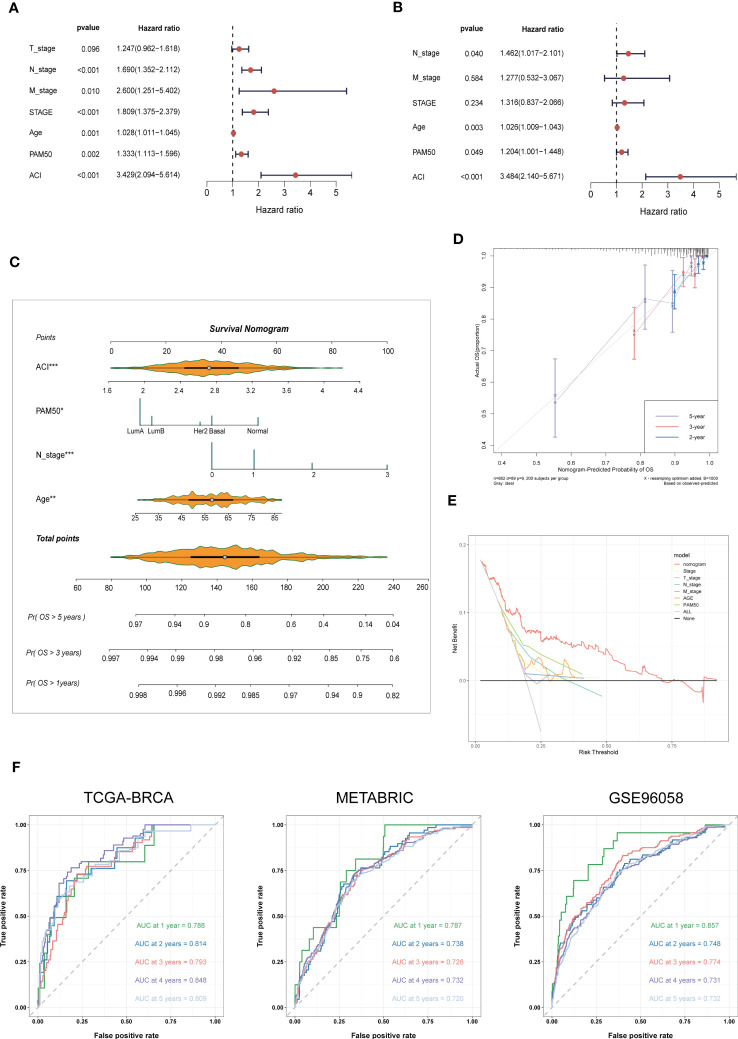
Nomogram Formulation Based on ACI Signature. **(A, B)** Univariate and Multivariate Cox regression analysis of the ACI signature and common clinical parameters in TCGA-BRCA. **(C)** Construction of a nomogram to predict OS of BC patients from TCGA-BRCA. **(D)** Nomograph-based correction plots were used to assess the agreement between predicted OS and actual OS. **(E)** The DCA curve was used to evaluate the clinical decision efficacy of the nomogram relative to other clinical indicators. **(F)** The ROC curves of nomogram in the TCGA-BRCA, METABRIC and GSE96058 datasets. separately. OS, overall survival; AUC, Area under the curve.

According to the univariate and multivariate Cox regression analyses performed on the TCGA-BRCA cohort, our ACI signature can serve as an independent predictor of prognosis for patients with BC (**Figures 6A, B**). According to the results, we incorporated the patients’ age, N stage, PAM50 subtype and ACI value to construct a clinicopathological nomogram for predicting individual OS at 1-, 3- and 5 years (**Figure 6C**). Furthermore, to verify the consistency of the nomogram predictions, the calibration plot was portrayed (**Figure 6D**). DCA curve in the training cohort indicated that the model could guide clinical application and be helpful for both OS and PFS of BC patients (**Figure 6E**). The time-dependent ROC curves were drawn to verify the sensitivity and specificity of the nomogram for predicting the survival time of patients with BC. The 1-, 3- and 5 year AUC value of the training set were 0.788, 0.793 and 0.809, respectively, and those of the METABRIC set and GSE96058 set were displayed in **Figure 6F**.

### 3.6 Gene set enrichment analysis and immune activity between two ACI groups

**Figure 7:**
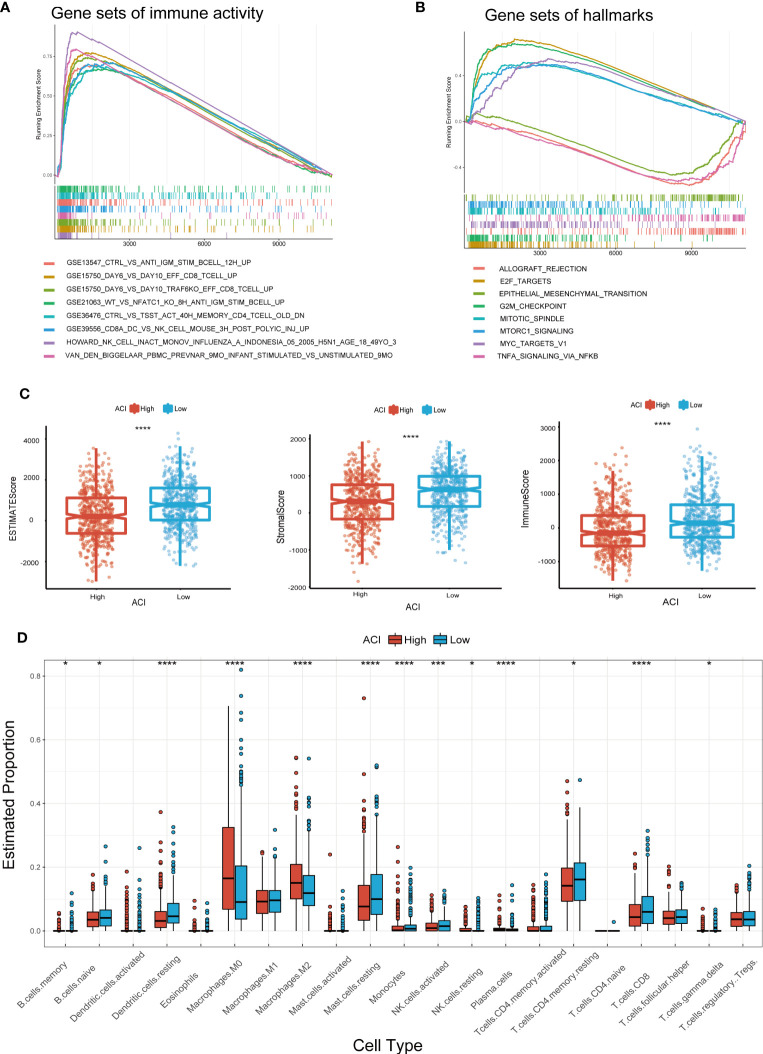
GSEA and Immune Activity Between Two ACI Groups. **(A, B)** Presentation of the top 8 differential pathways from C7 immune gene set **(A)** and Hallmark **(B)** of GSEA in TCGA-BRCA cohort. **(C)** Differences of ESTIMATE score, immune score, stromal score. **(D)** Boxplots were used to delineate the differences infiltration degree in 22 immune cells between two ACI groups patients. *p-value< 0.05; ***p-value< 0.001.

We use “GSVA” enrichment analysis in the TCGA-BRCA dataset to investigate the features of tumour immune activity and signal pathways between different ACI groups. The results of the C7 immune gene set indicated that multiple immune functions were enriched in the high ACI group (**Figures 7A**). The results of GSEA analysis using the KEGG database revealed significant enrichment of pathways such as “Allograft rejection,” “E2F targets,” “Epithelial-Mesenchymal transition,” “G2M checkpoint,” and TNFA signaling pathway in the group of patients with high ACI levels (**Figure 7B**).

Recent studies have shown that immune microenvironment plays a vital role in tumour progress and immunotherapy, therefore we focused on the TME landscape and characteristics of breast cancer patients in different ACI groups. The TME was evaluated and quantified through computing the interstitial score and immune score by ESTIMATE algorithm, and the results unrevealed that the interstitial score and immune score were lower in the high-ACI group (**Figure 7C**). Subsequently, we used the CIBERSORT Rpackage to quantify the relative proportions of infiltrating immune cells in different groups. From the results, we observed that macrophages M0 and M2 were gathered in the high-ACI group while CD8+ T cells, resting memory CD4+ T cells, monocytes, activated NK cells, resting mast cells and resting dendritic cells were significantly aggregated in the low-ACI group (**Figure 7D**).

### 3.7 Correlation between ACI and tumour-immune microenvironment

**Figure 8:**
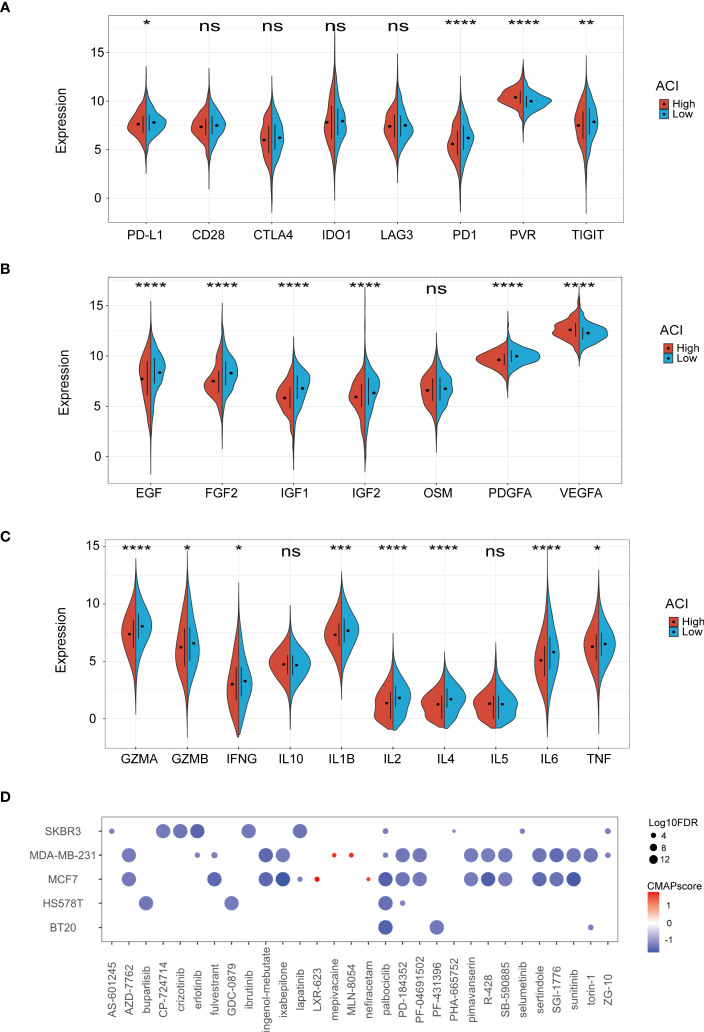
Correlation between ACI and TME. **(A-C)** Expression levels of immune checkpoints **(A)**, growth factor **(B)**, cytokines **(C)** between two ACI groups patients. **(D)** Potential clinical drugs identified targeted the high-ACI group BC by CMap. *p-value< 0.05; **p-value< 0.01; ***p-value< 0.001; ****p < 0.0001.

Considering the current immunotherapy drugs are mainly targeted at immunity checkpoints, we further discussed whether there is a difference in expression in the high -ACI and low ACI groups. It can be seen from the results that the PD1, PD-L1, and TIGIT of the low ACI group are higher, and the PVR expression in the high ACI group is more obvious (**Figure 8A**). Cytokines, as the core of the complex interaction between cancer cells and immune cells, can not only regulate tumour progress, but also affect the efficacy of immunotherapy. Some studies have shown that growth factors also affect the tumour immune microenvironment and the efficacy of immunotherapy. Consequently, we compared growth factors and cytokines expression levels between the two groups in TCGA-BRCA dataset. (**Figures 8B, C**). Recently, the CMap database has been applied for drug discovery in cancer research to locate potential treatment options for certain illnesses or conditions. By using the CMap database, we identified 30 compounds that exhibit the potential to serve as clinical agents for breast cancer in the high-ACI group (**Figure 8D**).

## 4 Discussion

Due to the heterogeneity of tumours, breast cancer patients remain at substantial risk of recurrence and death even after comprehensive treatment including surgery and adjuvant chemotherapy (37). Thus, the development of reliable prognostic molecular biomarkers could predict the likelihood of disease recurrence or progression in breast cancer patients and could even aid clinical decision making and improve clinical outcomes. As considerable anoikis, a type of programmed cell death in which cells separate from the appropriate extracellular matrix, was reported to regulate the biological behaviour of various tumours (38, 39). Recently, some studies have shown that anoikis related genes have a strong prediction ability for patients with different types of cancer (14, 15, 40). For breast cancer, anoikis resistance has been considered a biomarker with poor prognosis, which helps the formation or maintenance of breast cancer stem cell population (41, 42). However, few studies have been reported on the role of ARGs in breast cancer development and their impact on the immune microenvironment of BC. In our research, we constructed a prognostic signature based on ARGs, and explored its clinical guidance value, and further evaluated the TME of BC patients based on this prognostic signature to provide a reference for the field of anoikis in cancer research.

The prognostic signature constructed and trained in the TCGA-BRCA cohort consisted of 6 ARGs (CEACAM5, LAMB3, MAD2L1, PLK1, TP63 and PYCARD). Certain links between these genes and the pathogenesis as well as the development of tumours have been reported in previous researches. For example, Powell et al. indicated that CEACAM5 is considered a metastatic driver and its overproduction facilitates tumour outgrowth at metastatic sites by promoting mesenchymal-to-epithelial transition (43). Similarly, Laminin subunit beta-3 (LAMB3) encoding the β3 subunit of laminin-332, participating in the invasion and metastasis of colon cancer, pancreatic cancer, and prostate cancer (44, 45). We also found that low methylation levels at cg07168232 of LAMB3 were associated with poor prognosis in BC patients. Besides, overexpression of MAD2L1 leads to chromosomal instability in lung tumour cells, and is highly correlated with high levels of BRCA pathway activity and BRCA1/2 pathogenic mutations in breast cancer patients (46). Polo-like kinase 1 (PLK1) is frequently found overexpressed in a variety of tumours, participates in DNA damage response, autophagy, apoptosis and cytokine signal, and is associated with poor prognosis (47–49). Recently, a study suggested that TP63 is the maintenance of stem cell pluripotency and plays a unique inhibitory role in tumour progression by regulating cell cycle regulation, extracellular matrix remodel, epithelial-mesenchymal transition, and enrichment of pluripotent stem cells (50). However, the role of TP63 as a tumour promoter or tumour suppressor has been controversial (51). Furthermore, it has been demonstrated that the expression of PYCARD is correlated with both the response to tumor immunotherapy and prognosis. Our data indicated that elevated levels of PYCARD in BC are linked to improved overall survival (OS), although the precise mechanism is not yet clear and requires further investigation in future studies (52).

Recently, research modalities for establishing tumour prognostic signatures based on gene sets associated with specific biological features have often been reported. Borrowing from this approach, we constructed a breast cancer prognostic signature based on the expression of ARGs. ACI’s median value was used to classify BC patients into high and low groups. The analysis revealed a considerable difference in the OS of BC patients with high and low ACI levels across all datasets, indicating that the 6-gene signature is a reliable predictor of BC prognosis. Additionally, we developed a clinical prognostic nomogram incorporating ACI values, age, PAM50 subtype, and N stage of BC patients to predict their outcome. The DCA curve also showed that the nomogram was superior to a single independent clinical feature, which was helpful for clinical decision-making and benefited patients.

During the recent years, cancer immunotherapy has demonstrated robust anti-tumour effects in treating diverse types of cancers, including breast cancer. This type of therapy involves monoclonal antibody immune checkpoint inhibitors, therapeutic antibodies, cancer vaccines, cell therapy, and small molecule inhibitors (2). There is a close relationship between the heterogeneity of TME and the different response rates of tumour patients to immunotherapy (53). The classification of tumour immune microenvironment can better guide clinical treatment and achieve precision medicine. Thus, we explored the relationship between TME prospect estimation and ACI. As can be seen from the ESTIMATE results, the TME-related scores of the low-ACI group were higher than those of the high-ACI group, suggesting that ACI signature can be used as one of the indicators to identify patients with different TME, and the breast cancer in the high ACI group may have higher tumor purity. Besides, we also analysed the degree of immune cell infiltration, expression levels of immune checkpoints, growth factors and cytokines to further understand the differences of TME between the two groups. The clustering of macrophage M0 and M2 was noticeable in the high-ACI group, indicating an elevated likelihood of immune evasion by tumor cells in this group. Surprisingly, the proportion of anti-tumour immune cells, growth factors and cytokines in BC patients in the low-ACI group was higher, while high-ACI patients had a higher expression of VEGFA. VEGFA monoclonal antibodies and VEGFA receptor targeting drugs mediate immune stimulatory effects independent of ADCC and ADCP, largely reflecting VEGFA’s key role in establishing cancer-related immunosuppression (54). All these results suggest that BC patients in low-ACI group may have a more pronounced response and greater benefit to immunotherapy. Finally, we identified several potential therapeutic drugs for breast cancer patients in high-ACI group through CMap, including Palbociclib targeting CDK4/6 and Lapatinib targeting EGFR.

However, whether anoikis can positively affect the immune microenvironment of breast cancer remains unclear and requires further exploration.

## 5 Conclusion

In summary, our study identified a credible risk signature for BC patients based on anoikis-related transcriptomic profiling and performed systematic research of ARGs. This signature has been confirmed to independently predict the prognosis of breast cancer patients. The relationship between the ACI and TME implies anoikis-related genes could impact immunotherapy in specific populations. However, the underlying mechanisms, especially the function of anoikis on tumour immune infiltrating cells, remain unknown and need to be further explored.

## Data availability statement

The data that support the findings of this study are available in the public domain: The TCGA-BRCA dataset can be accessed through the Genomic Data Commons (GDC) data portal at https://portal.gdc.cancer.gov/. METABRIC project can be accessed through the European Genome-phenome Archive (EGA) at https://ega-archive.org/. The GSE96058 dataset can be accessed through the in the Gene Expression Omnibus (GEO) database at https://www.ncbi.nlm.nih.gov/geo/query/acc.cgi?acc=GSE96058.

## Author contributions

XL and QY contributed equally to this work. XL and WW designed the study. WW acquired the funding. XL, QY, CZ, SW and WW participated in data collection and analysis. QY, XL and SW wrote the original draft. CZ and WW read and revised the graphs and manuscript. All authors contributed to the manuscript and approved the submitted version.

## Supplementary material

Supplementary Figure 1 Heatmap and Kaplan-Meier plots of signature-included ARGs’ DNA methylation in BC generated using MethSurv. The red and blue lines indicate higher (β > cut-off) and lower (β < cut-off) methylation patient groups, respectively, dichotomized according to best cut-off point in MethSurv. HR, Hazard ratio; KIRC, Kidney renal clear cell carcinoma; KM, Kaplan–Meier; LR, Log-likelihood ratio.

Supplementary Figure 2 Correlation heatmaps of signature-included ARGs and clinicopathological features in datasets of METABRIC **(A)** and GSE96058 **(B)**.
