# Supplementary figures and images for: Predicting the immune microenvironment and prognosis with a anoikis - related signature in breast cancer
Source: Front Oncol. 2023 Jul 4;13:1149193. doi: 10.3389/fonc.2023.1149193 (PMC10353543; doi:10.3389/fonc.2023.1149193)

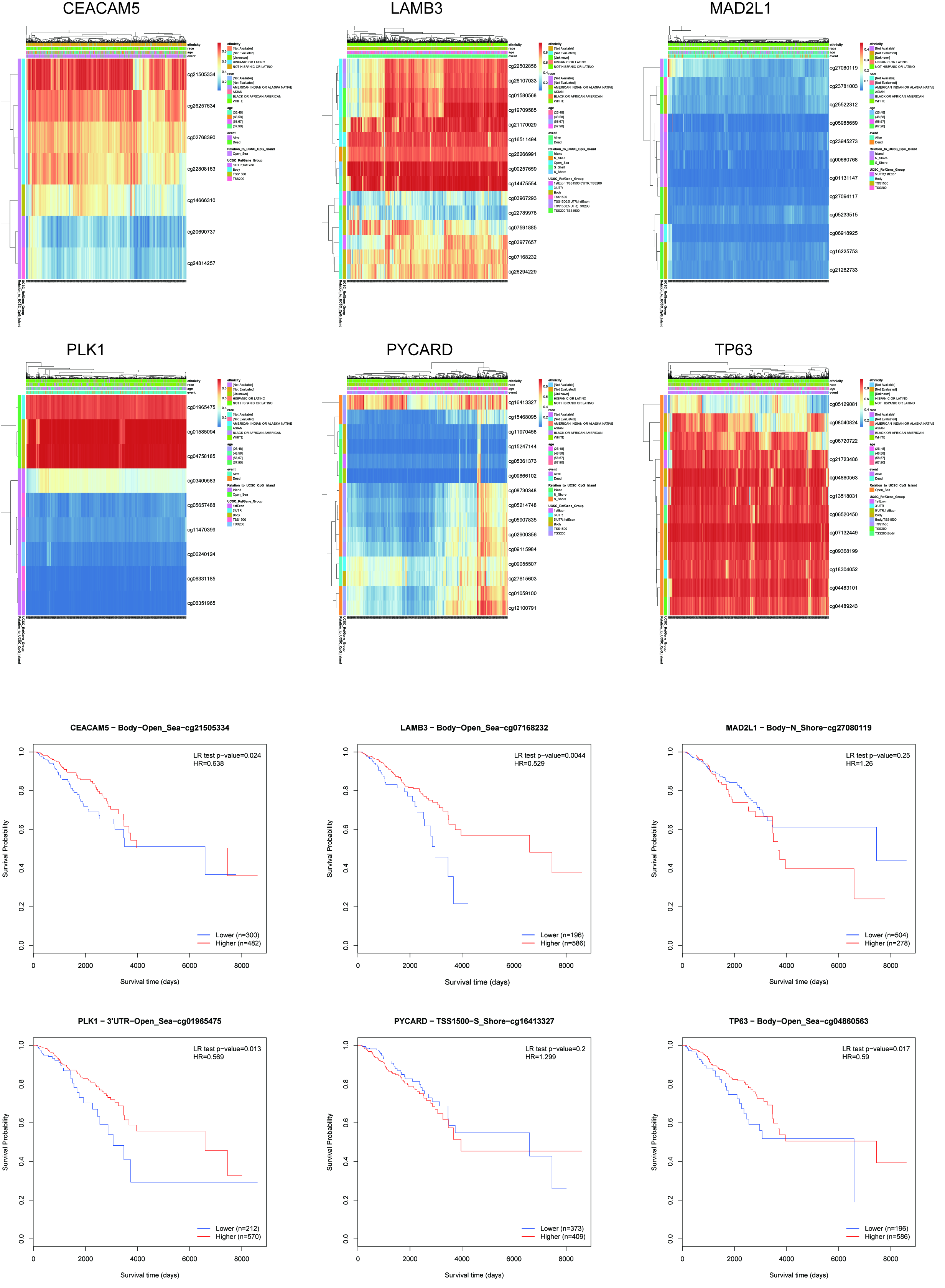

Supplement: Supplementary Figure 1 — Heatmap and Kaplan-Meier plots of signature-included ARGs’ DNA methylation in BC generated using MethSurv. The red and blue lines indicate higher (β > cut-off) and lower (β < cut-off) methylation patient groups, respectively, dichotomized according to best cut-off point in MethSurv. HR, Hazard ratio; KIRC, Kidney renal clear cell carcinoma; KM, Kaplan–Meier; LR, Log-likelihood ratio. [file Image_1.tif]

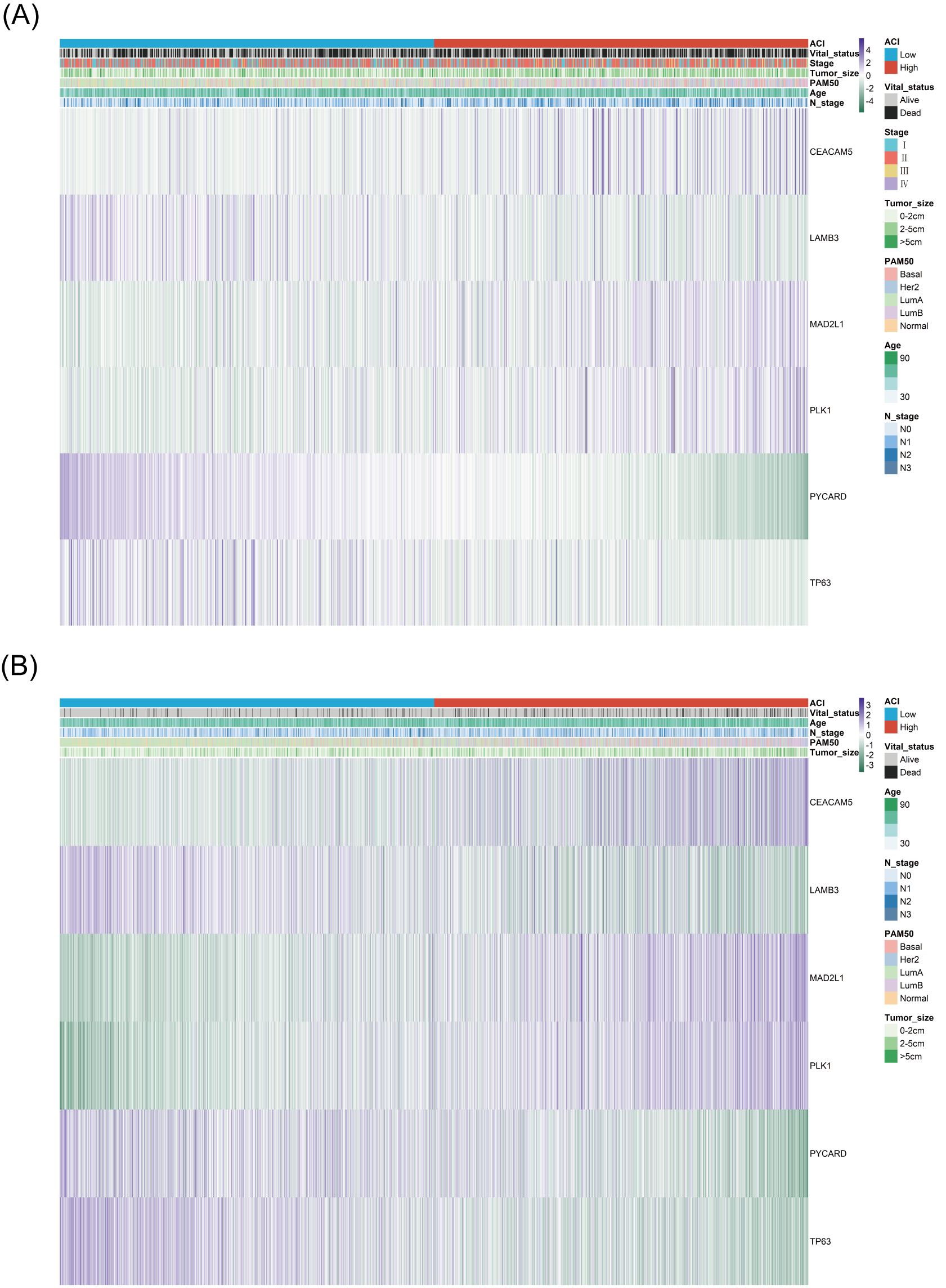

Supplement: Supplementary Figure 2 — Correlation heatmaps of signature-included ARGs and clinicopathological features in datasets of METABRIC (A) and GSE96058 (B). [file Image_2.tif]
