# Supplementary material for: Predicting the immune microenvironment and prognosis with a anoikis - related signature in breast cancer
Source: Front Oncol. 2023 Jul 4;13:1149193. doi: 10.3389/fonc.2023.1149193 (PMC10353543; doi:10.3389/fonc.2023.1149193)
Supplement: Supplementary file 3 [file Table_1.docx]

Supplementary Material

**SUPPLEMENTARY TABLE**

**Supplementary Table S1**. Primers sequences were shown.

| \| **Supplementary Table S1. Primers for qRT-PCR detection** \| \| \| \| --- \| --- \| --- \| \| CEACAM5 \| Forward \| GCCTCAATAGGACCACAGTCAC \| \| Reverse \| CAGGTTAAGGCTACAGCATCCTC \| \| LAMB3 \| Forward \| GTCACAGAGCAGGAGGTGGCT \| \| Reverse \| GCTTCTGTCAAGACTCTCCAGG \| \| MAD2L1 \| Forward \| TTGAGTGTGACAAGACTGCAAAAG \| \| Reverse \| CAGTGGCAGAAATGTCACCGTAG \| \| PLK1 \| Forward \| GCACAGTGTCAATGCCTCCAAG \| \| Reverse \| GCCGTACTTGTCCGAATAGTCC \| \| PYCARD \| Forward \| AGCTCACCGCTAACGTGCTGC \| \| Reverse \| GCTTGGCTGCCGACTGAGGAG \| \| TP63 \| Forward \| CAGGAAGACAGAGTGTGCTGGT \| \| Reverse \| AATTGGACGGCGGTTCATCCCT \| \| GAPDH \| Forward \| GTCTCCTCTGACTTCAACAGCG \| \| Reverse \| ACCACCCTGTTGCTGTAGCCAA \| |
| --- | --- | --- | --- | --- | --- | --- | --- | --- | --- | --- | --- | --- | --- | --- | --- | --- | --- | --- | --- | --- | --- | --- | --- | --- | --- | --- | --- | --- | --- | --- | --- | --- | --- | --- | --- | --- | --- | --- |
